# Mucin stimulates the growth of Legionella pneumophila

**DOI:** 10.1099/mic.0.001682

**Published:** 2026-03-02

**Authors:** Alexis Vargas, Hunter J. Kuhlemeier, Nicholas P. Cianciotto

**Affiliations:** 1 Department of Microbiology and Immunology, Northwestern University Medical School, Chicago, IL, USA

**Keywords:** chitinase, *Legionella pneumophila*, mucin, mucinase, protease, sliding motility, type II secretion system (T2SS)

## Abstract

Previously, it was demonstrated that a chitinase (ChiA) secreted by the *Legionella pneumophila* type II secretion system (T2SS) degrades mucin, thereby facilitating bacterial movement through a mucin layer. Here, we discovered that the addition of mucin to a chemically defined medium (CDM) greatly stimulates the growth of *L. pneumophila*, indicating that the bacterium can very effectively utilize mucin as a food source. This growth-stimulatory effect was evident in broth and agar media and for all WT strains tested. Remarkably, the growth of *L. pneumophila* on mucin-containing CDM agar rivalled the growth of the bacterium on buffered charcoal yeast extract (BCYE) agar, the standard medium used for cultivating legionellae. A *L. pneumophila* mutant lacking the T2SS was majorly impaired for growth in mucin-containing CDM, suggesting that exoenzymes are needed for mucin assimilation. In support of this hypothesis, mutants lacking either ChiA or the secreted protease ProA were also impaired for growth in the presence of added mucin. Finally, we observed that *L. pneumophila*, but not a mutant lacking secreted surfactant, exhibits a marked spreading phenotype (i.e. sliding motility) when grown on CDM agar vs. BCYE agar. However, WT bacteria grown on CDM agar containing mucin did not show this spreading, suggesting that sliding motility is induced under low-nutrient conditions but repressed by mucin byproducts. Taken together, these results provide new insight into the versatility of *L. pneumophila* physiology and suggest that *L. pneumophila* may grow well in extracellular spaces in the lungs that contain mucin.

## Introduction

The Gram-negative bacterium *Legionella pneumophila* is the most common cause of Legionnaires’ disease, a potentially fatal form of pneumonia that is occurring more frequently [1-3]. In the natural environment and in human-made water systems, *L. pneumophila* persists free-floating, within biofilms and as a parasite of amoebae [4,5]. Following delivery into the alveoli in the lower respiratory tract, *L. pneumophila* grows within macrophages, and then following lysis of these host cells, the amplified legionellae survive and spread extracellularly, resulting in further intracellular infections, inflammation and tissue destruction [4,6, 7]. In addition to the Dot/Icm type IV secretion system, the Lsp type II secretion system (T2SS) is a key virulence determinant of *L. pneumophila* [4,7, 8].

A subset of Gram-negative bacteria utilizes the T2SS for infection of larger hosts and/or for growth as free-living entities in the environment [9,10]. Substrates of the T2SS are initially transported across the bacterial inner membrane by the Sec or Tat translocon, and then, after attaining their tertiary form within the periplasm, enter the T2SS apparatus for transit across the bacterial outer membrane and delivery into the extracellular milieu [11,13]. Our work and that of others have demonstrated that the T2SS is important for *L. pneumophila* infection of multiple types of amoebae, biofilm formation, planktonic survival at low temperatures, sliding motility, growth in low-iron conditions, infection of macrophages and epithelial cells, dampening of cytokine secretion by infected host cells, degradation of pathogen-associated molecular patterns and host cytokines, infection of the murine lung, damage to lung tissue, resistance to human serum and resistance to polymyxin B, a cationic antimicrobial peptide/antibiotic [8,14,30]. We have identified as many as 120 substrates of the *L. pneumophila* T2SS [8,24, 25]. These secreted proteins encompass a wide array of degradative enzymes and many (novel) proteins that are unrelated to known proteins [8,19, 24, 25, 31,44]. Most of the characterized substrates of the *L. pneumophila* T2SS have homologues that are widely distributed across the 66 species in the *Legionella* genus [8,25].

One of the *L. pneumophila* T2SS substrates that is important for pathogenesis is chitinase (ChiA), which we first identified as a chitinase that also promotes bacterial survival in the murine lung [35]. We posited that ChiA is a bi-functional enzyme that acts on a chitin-like factor in the infected alveolar space and whose degradation aids *L. pneumophila*. Subsequent analysis of purified ChiA revealed a C-terminal domain that has a chitinase active site as well as a peptidase active site that mediates the cleavage of mucins [42]. Expressed in the lungs and elsewhere, mammalian mucins are high-molecular-weight glycoproteins that contain large numbers of heavily *O*-glycosylated Ser/Thr-rich repeat sequences [45,48]. These mucins exist as cell surface-exposed transmembrane proteins or secreted gel-forming proteins, which create a mucosal barrier that acts as a front-line defence against infection [45,49,51]. In line with the activity of ChiA, a *L. pneumophila chiA* mutant is impaired for the ability to penetrate a mucin layer, potentially explaining the role of ChiA *in vivo* [42]. Since mucinases from lung pathogens or intracellular parasites have been minimally studied, especially relative to those from enteric microbes [52,53], we sought to investigate ChiA-mediated migration through mucin as a possible step in *L. pneumophila* infection of alveolar macrophages or epithelia. Unexpectedly, however, we found that mucin greatly stimulates the extracellular replication of *L. pneumophila* and therefore proceeded to investigate the role of the T2SS in that mucin utilization.

## Methods

### Bacterial strains

*L. pneumophila* WT strain 130b [American Type Culture Collection (ATCC) strain BAA-74] was previously described, as were its *lspF* mutant NU275, complemented NU275, *chiA* mutant NU318, *proA* mutant AA200, *pilE* mutant BS100, *flaA* mutant NU348, *flaA pilE* mutant NU350 and *bbcF* mutant NU396 [15,17, 35, 54]. *L. pneumophila* WT strains Paris (Collection de l’Institut Pasteur strain 107629), Philadelphia-1 (ATCC 33152) and Togus-1 (ATCC 33154) were also previously described [55,57], as were strains of other *Legionella* species, i.e. *Legionella anisa* WA 316-C3 (ATCC 35292), *Legionella bozemanii* WIGA (ATCC 33217), *Legionella cardiaca* H63^T^ (ATCC BAA-2315), *Legionella dresdenensis* WO3-356 (German Collection of Microorganisms 19488), *Legionella feeleii* WO-44C (ATCC 35072), *Legionella lansingensis* 1677-MI-H (ATCC 49751), *Legionella londiniensis* 1477 (ATCC 49505), *Legionella longbeachae* Long Beach 4 (ATCC 33462), *Legionella micdadei* Stanford-M, *Legionella spiritensis* MSH-9 (ATCC 35249), *Legionella wadsworthii* 81–716A (ATCC 33877) and *Legionella worsleiensis* 1347 (ATCC 49508) [54,58,67].

### Bacteriological media

*L. pneumophila* strains were routinely grown on buffered charcoal yeast extract (BCYE) agar [68]. In some cases, as indicated below, bacteria were also grown on a variant of BCYE agar in which the customary ferric pyrophosphate supplement was omitted, resulting in a medium (BCYE – Fe supplement) that had ~7 µM residual iron [69]. To assess the impact of mucin on *L. pneumophila* growth, bacteria were grown either in chemically defined medium (CDM) broth or on CDM agar that had mucin added. For the liquid medium, the CDM base was prepared as before [24], except for the iron supplement being set at 7 µM ferric pyrophosphate. As usual, the CDM base broth was filter-sterilized. The mucin stock that was used to supplement the CDM base was freshly prepared by adding 1 g of mucin solid (i.e. type III mucin from Sigma-Aldrich, catalogue #M1778-10G) to 25 ml of the CDM broth (in a 100-ml bottle), stirring the slurry for 5 min and then, as before (70), autoclaving for 15 min. For the CDM agar medium, 2.5 g of mucin solid (as above), 1.5 g of activated charcoal (Sigma-Aldrich, catalogue #C4386-2.5KG) and 15 g of agar (Research Products International, CAS #9002-18-0) were added to 1 l of CDM base broth (in a 2-l flask), and then, the mixture was autoclaved for 20 min. The ferric pyrophosphate supplement (as above) was added after the autoclaved media had cooled in a water bath. The CDM+mucin agar plates were used within a week of being made.

### Assays for *L. pneumophila* growth in the presence of mucin

Legionellae that had been grown on standard BCYE agar were suspended in PBS to an OD_660_=0.3 and then diluted 1,000-fold in PBS, resulting in a suspension of ~1×10^6^ c.f.u. ml^−1^. Three-hundred microlitres of the cell suspension were added to 2.7 ml of CDM broth that contained either 0, 2.5, 5 or 10 mg ml^−1^ mucin added. These mucin concentrations were initially chosen because they (i) approximated the range of mucin concentrations in human lungs and (ii) kept our experiments in line with earlier studies that had shown a stimulatory effect of mucin on the *in vitro* growth of other bacteria [46,70,75]. The samples (*n*=3, per strain tested), which were in 14-ml polypropylene tubes and had starting inocula equal to ~1×10^5^ c.f.u. ml^−1^, were then incubated at 37 °C with shaking. At *t*=0, 4, 24 or 48 h, 20-µl aliquots were removed and subjected to 10-fold serial dilution in PBS. Then, 20-µl aliquots from the undiluted samples and each of the dilutions were plated onto BCYE agar to obtain the starting and surviving c.f.u. ml^−1^. Thus, based on the above protocol, the lower level of detection for surviving bacteria was ~50 c.f.u. ml^−1^. Since 2.5 mg ml^−1^ of mucin did not appear to stimulate *L. pneumophila* growth in CDM broth in preliminary trials, we completed this set of experiments by comparing the effects of 0, 5 and 10 mg ml^−1^ mucin added. To test the impact of mucin on growth on a solid medium, strains that had been grown on standard BCYE agar were resuspended in PBS to an OD_660_=0.3 and then subjected to 10-fold serial dilutions in PBS. Five-microlitre aliquots taken from the dilution series were spotted onto CDM agar containing 0 or 2.5 mg ml^−1^ mucin, and the plates were sealed with parafilm and incubated at 37 °C. As a control, the bacteria were also spotted onto BCYE – Fe supplement agar. Alternatively, 100-µl aliquots were spread onto the entire surface of the CDM agar and BCYE agar and then incubated. Images were taken of the areas of bacterial growth, following 3–9 days of incubation at 37 °C.

### Statistical methods

Quantitative assays utilized three technical replicates, and the values obtained were given as the means and standard deviations. *P* values were determined by the Student’s t-test. For biological replicates, all experiments were repeated on at least three occasions, as indicated in the figure legends.

## Results

### Mucin greatly stimulates the growth of *L. pneumophila*

**Fig. 1.**
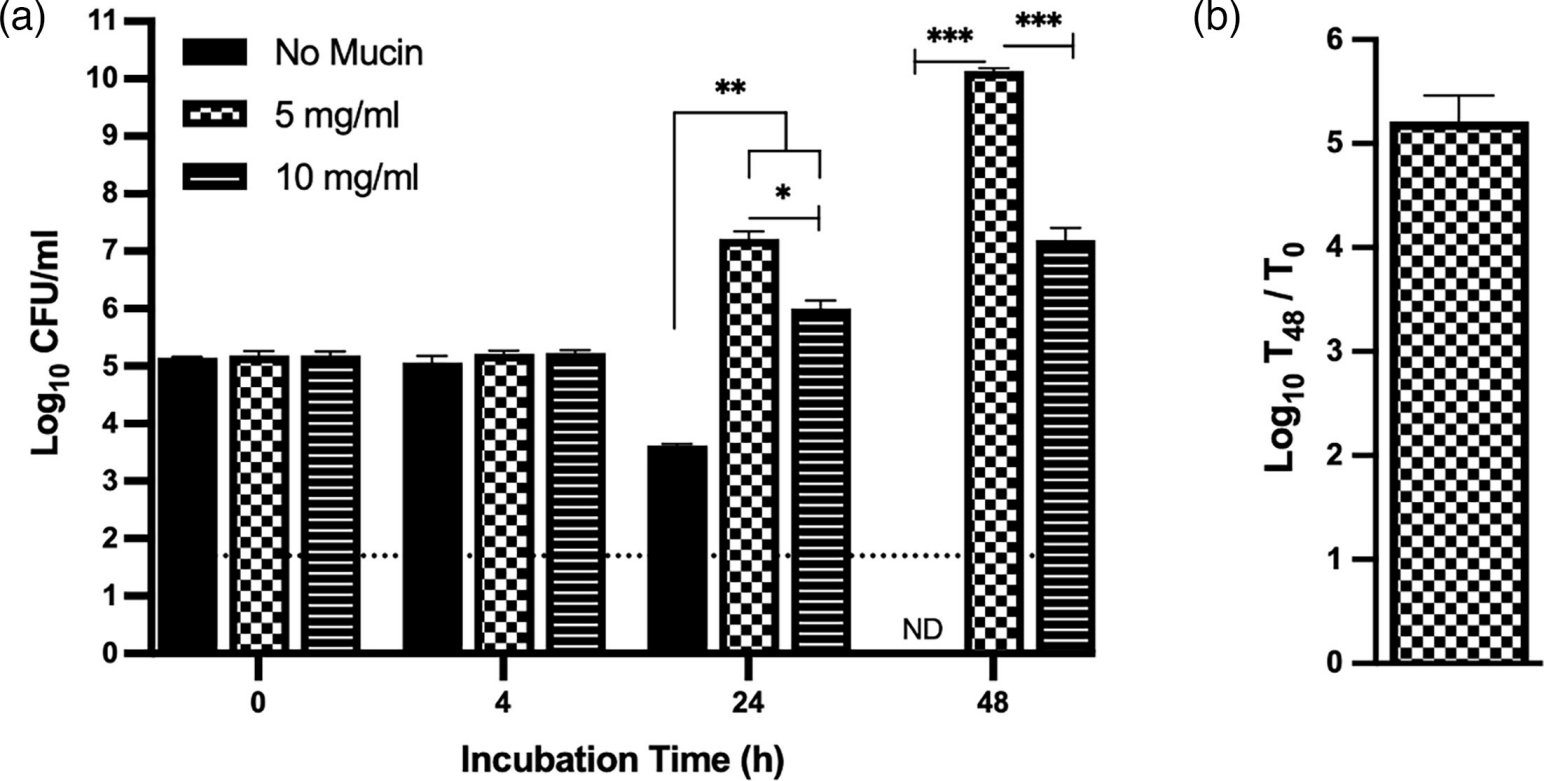
Effect of mucin on *L. pneumophila* strain 130b growth in CDM broth. Following growth on BCYE agar for 3 days at 37 °C, WT 130b was resuspended to 1×10^5^ c.f.u. ml^−1^ in CDM broth containing either no added mucin (black bars), 5 mg ml^−1^ mucin (checker-filled bars) or 10 mg ml^−1^ mucin (line-filled bars). Three-millilitre aliquots of the samples were added to tubes, and the suspensions were incubated with shaking at 37 °C. At the indicated time points, bacterial numbers were determined by plating for c.f.u. on BCYE agar. Values presented are the mean and standard deviations from three technical replicates. ‘ND’ indicates when no c.f.u. were recovered, with the dashed line denoting the level of detection possible. Asterisks indicate when different levels of c.f.u. were detected; *P<0.05; **P<0.01; ***P<0.001. For comparisons at the 48 h time point, the c.f.u. value for the no-mucin sample was conservatively set at the level of detection. The data presented here are representative of the results obtained from at least three independent experiments. (**b**) The average fold-increase (in log-10 units) in 130b c.f.u. at *t*=48 h when incubated in CDM broth containing 5 mg ml^−1^ mucin, using the data pooled from three experiments.

**Fig. 2.**
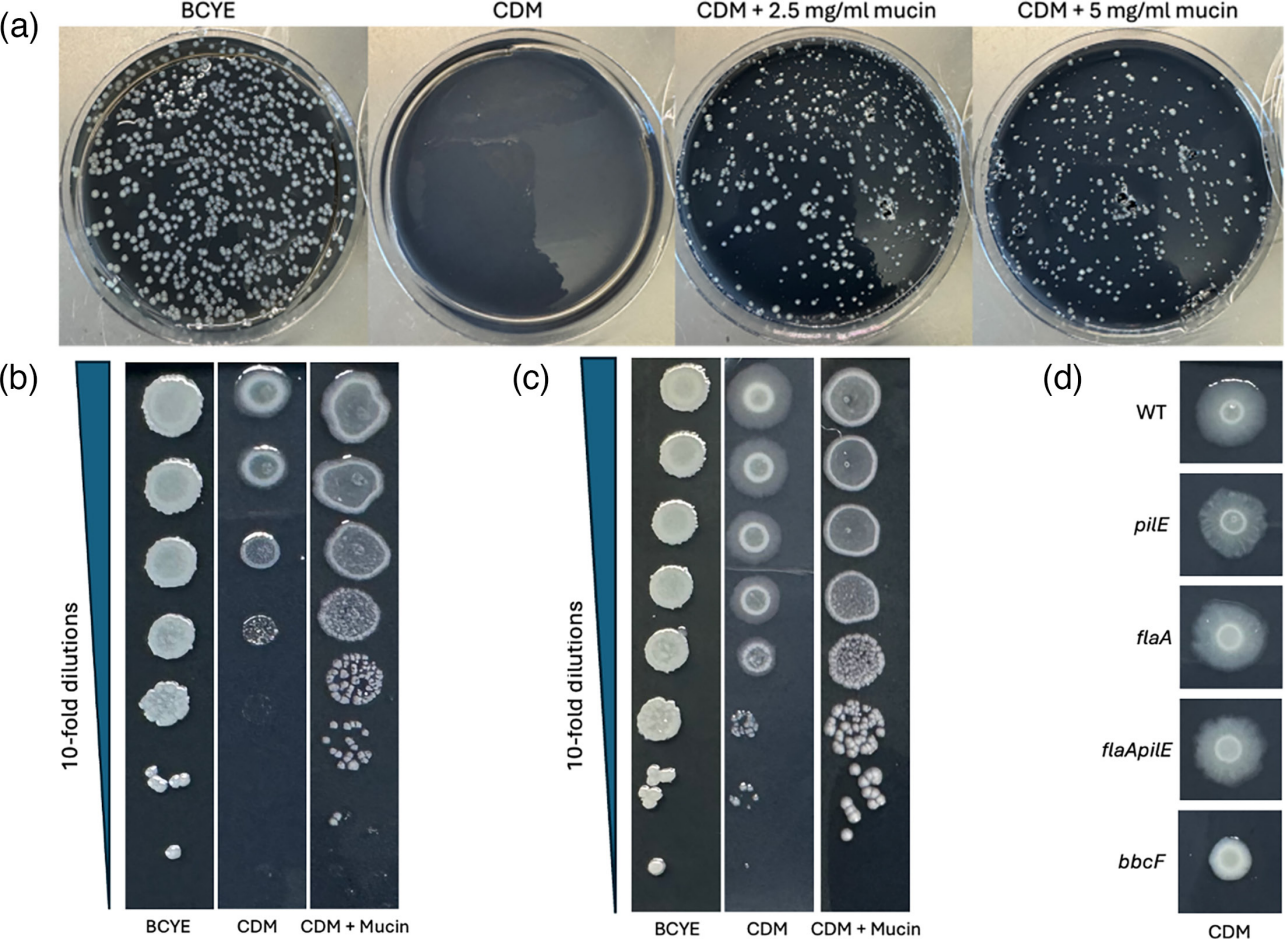
Effect of mucin on *L. pneumophila* strain 130b growth on CDM agar. (**a**) After growth on BCYE agar for 3 days at 37 °C, WT 130b was resuspended in PBS to an OD_660_=0.3, diluted 10^6^-fold, and then identically sized aliquots were plated for c.f.u. on either BCYE agar, CDM agar or CDM agar containing mucin added at 2.5 mg ml^−1^ or 5 mg ml^−1^. Images of bacterial growth were taken after 9 days of incubation at 37 °C. (b–c) Following growth on BCYE agar, as noted in (**a**), 130b bacteria were subjected to 10-fold serial dilutions in PBS, and then aliquots were spotted onto either BCYE agar, CDM agar or CDM agar containing 2.5 mg ml^−1^ mucin. Images of bacterial growth were obtained after 4 days (**b**) or 6 days (**c**) of incubation at 37 °C. (**d**) Following growth on BCYE agar for 3 days at 37 °C, WT 130b (WT), *pilE* mutant (*pilE*), *flaA* mutant (*flaA*), *flaA pilE* mutant (*flaA pilE*) and *bbcF* mutant (*bbcF*) were resuspended in PBS to an OD_660_=0.3, and then aliquots were spotted onto CDM agar. Images of bacterial growth were obtained after 6 days of incubation at 37 °C. For panels (a–d), the results presented here are representative of the results from at least three independent experiments.

While adapting the mucin-penetration assay to its more versatile syringe format [76], which meant incubating the legionellae in the presence of mucin for longer times, it appeared that *L. pneumophila*’s growth might be stimulated by the presence of mucin. Although a variety of unrelated and mostly enteric bacteria are known to use mucin for growth [52,70, 73, 77,79], there had been no prior reports of *L. pneumophila* utilizing (any form of) mucin as a nutrient or growth-stimulatory signal. Thus, we shifted focus to discern if mucin indeed promotes *L. pneumophila* growth. We assessed the ability of added mucin to enhance WT strain 130b growth in CDM broth. The CDM base consisted of 20 amino acids (with cystine replacing cysteine), *α*-ketoglutarate, glutathione, pyruvate, 10 metals (with the iron component set at 7 µM ferric pyrophosphate), KH_2_PO_4_, NaCl and 3-(N-morpholino) propanesulfonic acid buffer [24]. The mucin supplement used here included MUC1 and MUC5AC, which are two of the major mucins expressed in the airways and lungs [46,48, 80, 81]. When inoculated into the CDM base broth at a starting dose of ~1×10^5^ c.f.u. ml^−1^, the legionellae did not grow, but rather the numbers of c.f.u. declined and at 48 h were often undetected (Fig. 1a). However, the addition of mucin at 5 mg ml^−1^ dramatically stimulated growth of the bacteria, with there being a 100-fold or more increase by 24 h and a ~1,000-fold further increase by 48 h (Fig. 1a, b). Growth stimulation also occurred when mucin was added at 10 mg ml^−1^ (Fig. 1a). However, the lower c.f.u. ml^-1^ achieved in this case (relative to that obtained in the samples with 5 mg ml^−1^ mucin) suggested that mucin or mucin breakdown products might be inhibitory at high concentrations and that the bacterium’s ability to properly degrade or process the greater amount of mucin is insufficient under these growth conditions. As an alternate assay, we incorporated CDM into an agar format, which, to our knowledge, had never been done before. The growth-stimulatory effect of mucin was again plainly evident, whether we plated a single dilution of WT 130b for isolated colonies (Fig. 2a) or spotted aliquots from a set of serial dilutions (Fig. 2b). Moreover, the growth on CDM agar containing 2.5 mg ml^−1^ of mucin rivalled that seen on BCYE agar, the rich agar-based medium that is the long-time standard in the *Legionella* field (Fig. 2a, b).

Interestingly, when the 130b bacteria spotted onto the agar media were allowed to incubate for longer periods of time, growth on the CDM agar produced a zone of ‘spreading’ emanating from the central area of growth that was not evident during growth on the traditional BCYE agar (Fig. 2c). This spreading did not occur on the CDM agar containing added mucin (Fig. 2c). Thus, the CDM medium appeared to trigger a form of motility that was repressed by the presence of mucin. *L. pneumophila* exhibits three forms of motility, i.e. flagella-mediated swimming, type IV pilus-mediated twitching motility and surfactant-mediated sliding motility [17,54, 82,86]. Hence, we tested mutants of strain 130b lacking one or more of these systems, i.e. a *pilE* mutant lacking pilin, a *flaA* mutant lacking flagellin, a *flaA pilE* mutant missing both pilin and flagellin and a *bbcF* mutant lacking a biosynthetic enzyme (ketoacyl-acyl carrier protein synthase III) for surfactant production [17,54, 86]. The spreading zone was absent for the *bbcF* mutant that lacks surfactant and its associated sliding motility (Fig. 2d).

**Fig. 3.**
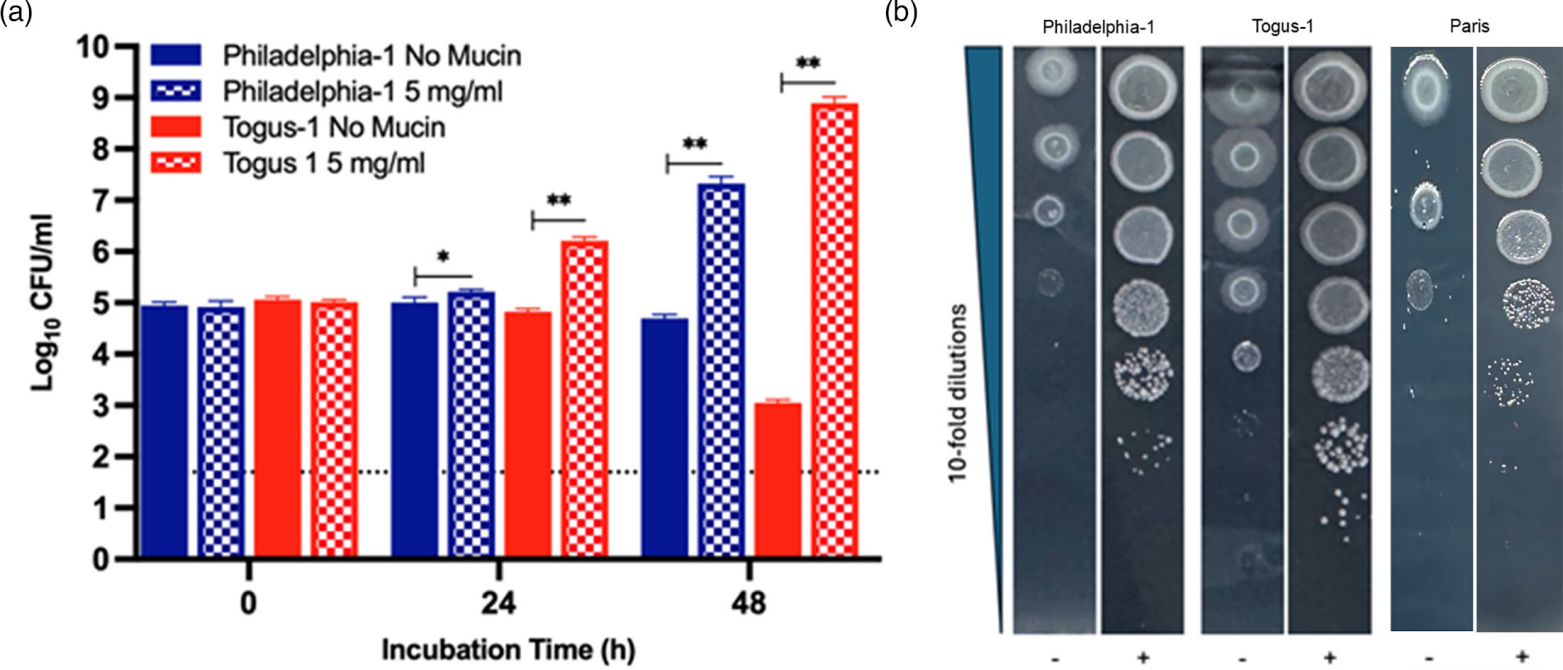
Effect of mucin on the growth of *L. pneumophila* strains Philadelphia-1, Togus-1 and Paris. Following growth on BCYE agar for 3 days at 37 °C, WT Philadelphia-1 (blue bars) and Togus-1 (red bars) were resuspended to ~1×10^5^ c.f.u. ml^−1^ in CDM broth containing either no added mucin (solid bars) or 5 mg ml^−1^ mucin (checker-filled bars) and then incubated with shaking at 37 °C. At the indicated time points, bacterial numbers were determined by plating for c.f.u. on BCYE agar. Values presented are the mean and standard deviations from three technical replicates, with the dashed line denoting the level of detection possible. Asterisks indicate increased levels of c.f.u. in the samples containing added mucin; *P<0.05; **P<0.01. (**b**) After growth on BCYE agar for 3 days at 37 °C, Philadelphia-1, Togus-1 and Paris were subjected to 10-fold serial dilutions in PBS, and then aliquots of the samples were spotted onto CDM agar without added mucin (−) or CDM agar containing 2.5 mg ml^−1^ mucin (+). Images of bacterial growth were obtained after 4 days of incubation at 37 °C. For both (**a**) and (**b**), the data presented are representative of the results obtained in at least three independent experiments.

*L. pneumophila* strains Philadelphia-1 and Togus-1 also showed enhanced growth in the presence of mucin, whether examined in the broth-based assay or the agar-based assay (Fig. 3a, b). Further tests using the agar-based assay revealed that *L. pneumophila* strain Paris also grows better in the presence of added mucin (Fig. 3b). Thus, mucin utilization was not an oddity of strain 130b but appears to be a trait common to *L. pneumophila* strains. Strain Togus-1 also showed marked surface-spreading that was repressed by mucin (Fig. 3b). Taken together, these results indicated that *L. pneumophila* not only degrades mucin [42] but uses its breakdown products as a food source. Moreover, in the absence of mucin, *L. pneumophila* may spread more easily via sliding motility.

### Mucin utilization by other *Legionella* species

**Fig. 4.**
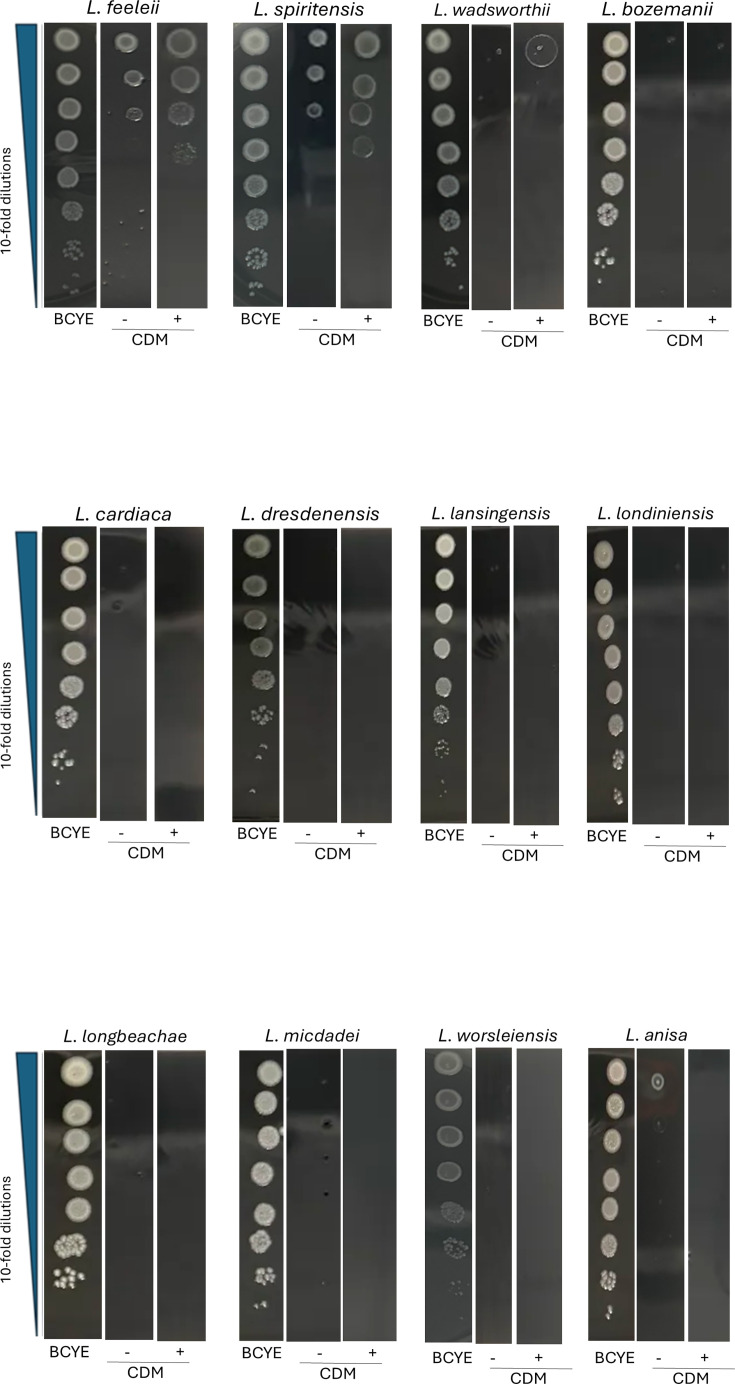
Effect of mucin on the growth of other *Legionella* species. After growth on BCYE agar for 3 days at 37 °C, strains representing the indicated *Legionella* species were subjected to 10-fold serial dilutions in PBS, and then aliquots of the samples were spotted onto either BCYE agar, CDM agar without added mucin (−) or CDM agar containing 2.5 mg ml^−1^ mucin (+). Images of bacterial growth were obtained after 4 days of incubation at 37 °C, and there was evidence of increased growth when the plates were reexamined at day 6. The data presented are representative of the results obtained from three independent experiments.

There are >67 *Legionella* species in addition to *L. pneumophila* [87]. To determine if mucin utilization is a trait shared among the other legionellae, we tested strains representing 12 of the other *Legionella* species for their growth on mucin-containing media. *L. feeleii*, *L. spiritensis* and, to a lesser degree, *L. wadsworthii* grew better on mucin-containing CDM agar than on non-mucin-containing CDM agar (Fig. 4), showing that they, like *L. pneumophila*, can utilize mucin. In contrast, *L. bozemanii*, *L. cardiaca*, *L. dresdenensis*, *L. lansingensis*, *L. londiniensis*, *L. longbeachae*, *L. micdadei* and *L. worsleiensis* did not grow on either the mucin-containing CDM or the non-mucin-containing CDM agar (Fig. 4), suggesting that they lack the capacity to utilize mucin, although it is formally possible that there is a factor lacking and/or inhibitory in the CDM base that mucin cannot overcome. Finally, *L. anisa*, although growing slightly on the CDM agar, did not exhibit any growth in the presence of mucin (Fig. 4), indicating that it too is unable to utilize mucin. Thus, mucin utilization appears not to be a trait conserved across the *Legionella* genus and may be limited to a relatively small subset of species, which includes, at least, *L. pneumophila, L. feeleii* and *L. spiritensis*, and perhaps *L. wadsworthii*.

### The T2SS promotes *L. pneumophila* growth in mucin-containing media

**Fig. 5.**
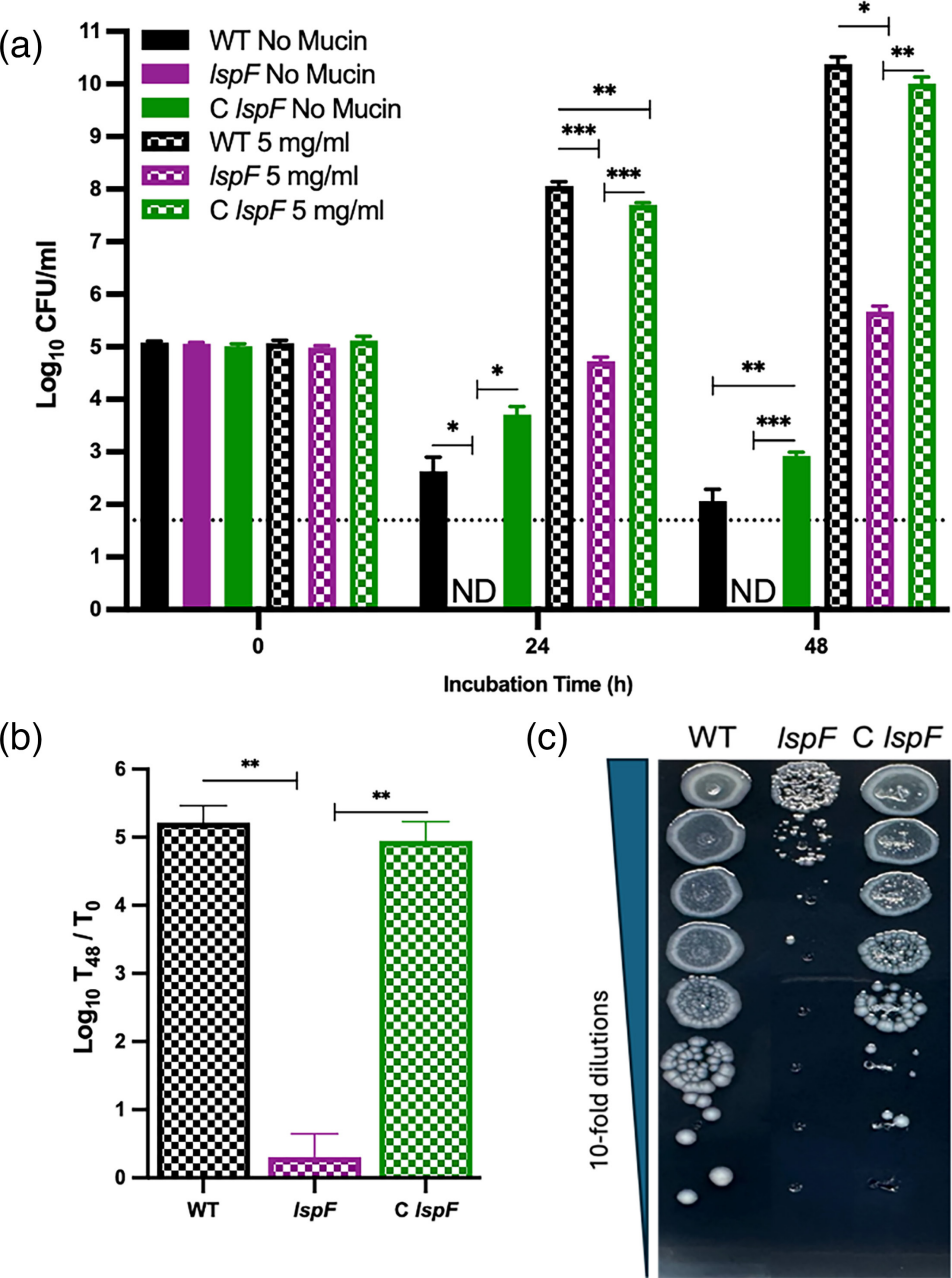
Growth of *L. pneumophila* WT, *lspF* mutant and complemented *lspF* mutant on mucin-containing CDM media. (**a**) Following growth on BCYE agar for 3 days at 37 °C, WT strain 130b (WT, black bars), *lspF* mutant (*lspF,* purple bars) or a genetically complemented *lspF* mutant (C *lspF*, green bars) were suspended in CDM broth containing no added mucin (solid bars) or 5 mg ml^−1^ mucin (chequered bars) and then incubated with shaking. At the indicated times, samples were plated for c.f.u. on BCYE agar. Values presented are the mean and standard deviations from three technical replicates, and the data presented here are representative of the results obtained from three independent experiments. ‘ND’ indicates when no c.f.u. were detected, with the dashed line denoting the level of detection possible. Asterisks indicate when there were differences in c.f.u. between samples; *P<0.05; **P<0.01; ***P<0.001. For these comparisons, the c.f.u. values for the no-mucin samples were conservatively set at the level of detection. (**b**) The average fold-increase (in log-10 units) in WT, *lspF* mutant and complemented mutant c.f.u. at *t*=48 h when incubated in CDM broth containing 5 mg ml^−1^ mucin, using the data pooled from three experiments. Asterisks indicate the difference between the *lspF* mutant and WT and complement; **P<0.01. (**c**) Following growth on BCYE agar, as noted in (**a**), WT, *lspF* mutant and complemented mutant were subjected to 10-fold serial dilutions in PBS, and then aliquots were spotted onto CDM agar containing 2.5 mg ml^−1^ mucin. Images of bacterial growth were obtained after 4 days of incubation at 37 °C. The data presented are representative of three independent trials.

As noted in the Introduction, the *L. pneumophila* T2SS secretes a variety of defined and putative proteases, peptidases and carbohydrate-degrading enzymes that could act on mucin glycoproteins [8,25, 42]. Therefore, to begin to discern the basis for *L. pneumophila*’s growth on mucin, we tested a mutant (*lspF*) lacking a functional T2SS apparatus for its ability to grow in mucin-supplemented CDM broth. Like its WT parent (Fig. 1a), the *lspF* mutant exhibited a loss in recoverability when inoculated at ~1×10^5^ c.f.u. ml^−1^ into base CDM broth (Fig. 5a). Although the mutant’s loss was greater than that of WT at 24 h, the difference in recoverability between the strains was ambiguous at 48 h, since, at that time point, WT recovery was just above the limit of detection, and the mutant was not recovered. When the *lspF* mutant was inoculated into CDM broth containing added mucin, it fully survived (relative to its starting c.f.u.) but, unlike parental WT, did not show any increase in c.f.u. at 24 h (Fig. 5a). Moreover, the mutant barely increased in numbers between 24 h and 48 h post-inoculation (Fig. 5a). Thus, whereas the difference between WT and mutant in the base CDM broth at 48 h was ~100-fold at the most and ~2-fold at the least, the difference between WT and mutant in the mucin-containing CDM broth at 48 h was ~10^5^-fold (Fig. 5a, b). A complemented mutant carrying a restored *lspF* gene behaved like the WT did (Fig. 5a, b). The impaired ability of the *lspF* mutant, but not its complement, to grow in the presence of mucin was also seen when the bacteria were plated onto mucin-containing CDM agar (Fig. 5c). Importantly, the *lspF* mutant (and other T2SS mutants) does not have a generalized growth defect in other nutrient-rich media, e.g. BCYE agar or buffered yeast extract broth, which is the standard liquid medium in the *Legionella* field [15,88]. Taken together, these data indicated that the T2SS promotes the growth of *L. pneumophila* in mucin-containing CDM. We hypothesize that this effect of the T2SS is mainly due to the ability of *L. pneumophila* exoenzymes to degrade the mucin in the media and thereby provide nutrients for growth, with the remainder of the effect linked to increased bacterial survivability in the CDM base. In support of this hypothesis are the known impact of T2SSs on mucin degradation by various other bacteria and the plethora of degradative enzymes secreted by the *L. pneumophila* T2SS [8,9, 25, 42, 89,91].

### Mutants lacking the T2SS substrates ChiA and ProA display impaired growth on mucin

**Fig. 6.**
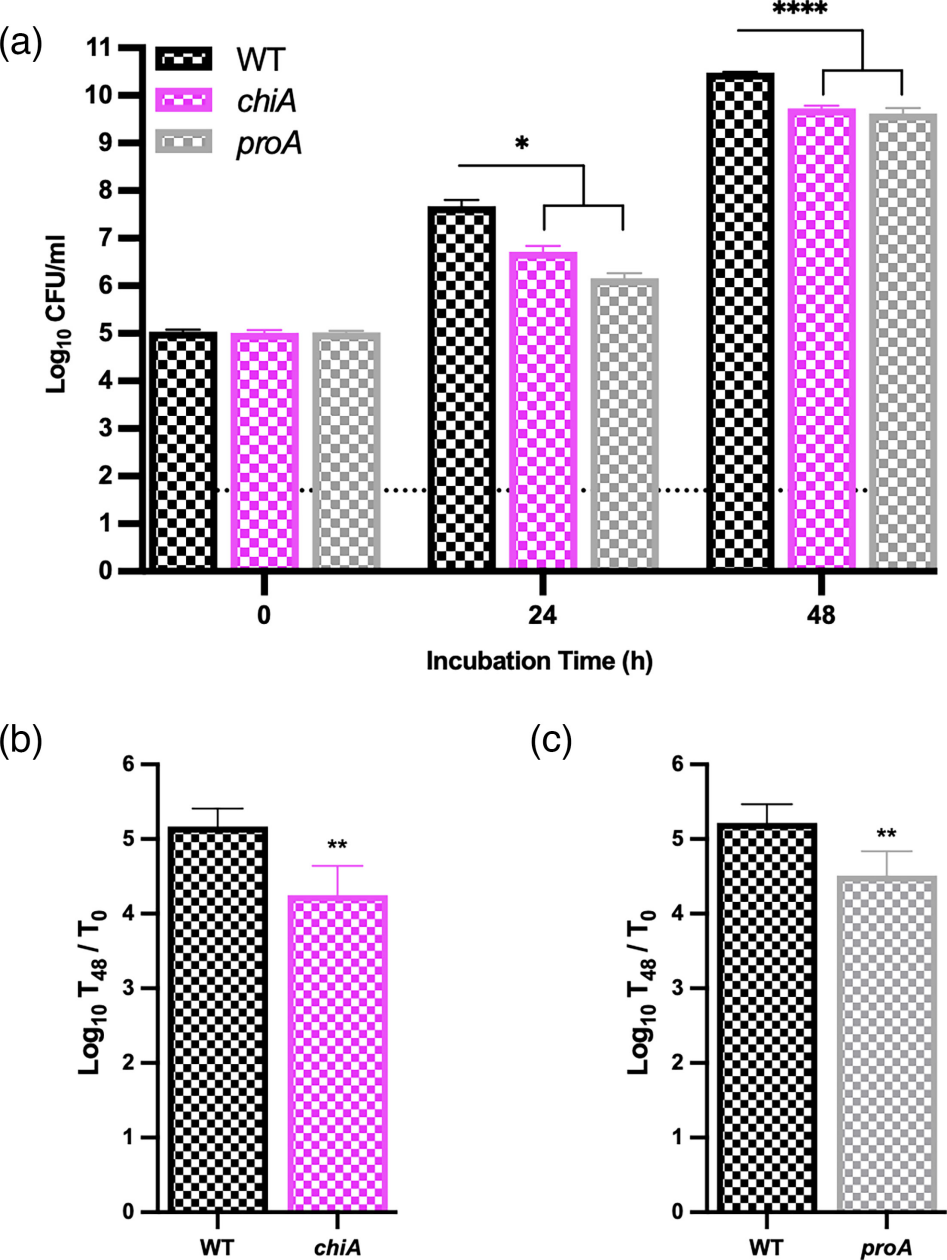
Growth of *L. pneumophila* WT, *chiA* mutant and *proA* mutant in mucin-containing CDM broth. (**a**) After growth on BCYE agar at 37 °C, WT 130b (WT, black chequered bars), *chiA* mutant (*chiA*, pink chequered bars) and *proA* mutant (*proA*, grey chequered bars) were inoculated into CDM broth containing 5 mg ml^−1^ mucin and then incubated at 37 °C. At the indicated time points, bacterial numbers were determined by plating. Values presented are the mean and standard deviations from three technical replicates, with the dashed line denoting the level of detection possible. Asterisks indicate when there were differences between WT and the mutants; *P<0.05; ****P<0.0001. The data presented here are representative of the results obtained from three experiments. (**b**) Average fold-increase (in log-10 units) in WT and *chiA* mutant c.f.u. at *t*=48 h, using the data pooled from three experiments. Asterisks indicate the difference in growth between the strains; **P<0.01. (**c**) Average fold-increase (in log-10 units) in WT and *proA* mutant c.f.u. at *t*=48 h, using the data pooled from three experiments. Asterisks indicate the difference in growth between the strains; **P<0.01.

Given that ChiA is a substrate of the *L. pneumophila* T2SS and that purified ChiA degrades mucin [25,35, 42], we tested a *chiA* mutant for its ability to utilize mucin for growth. Although the mutant grew within the first 24 h of incubation, its numbers were ~10-fold lower than those of parental WT (Fig. 6a). A similar pattern was observed at the 48 h time point (Fig. 6a, b), implying that ChiA promotes optimal utilization of mucin as a food source. Since the *chiA* mutant was not nearly as impaired as the *lspF* mutant was (Fig. 5), we posited that more T2SS-dependent enzymes act on the mucin glycoprotein. One of the most-characterized T2SS substrates of *L. pneumophila* is ProA, a metalloprotease that is abundantly expressed in *L. pneumophila* cultures and promiscuous in its range of protein targets [8,29]. When a mutant specifically lacking the T2SS-dependent ProA was tested, it exhibited a ~10-fold reduced ability to grow in the mucin-containing CDM (Fig. 6a, c), suggesting that ProA also promotes the utilization of mucin. Overall, these data indicated that the ChiA and ProA proteins secreted by the T2SS help degrade mucin into a form that is assimilated by *L. pneumophila* for growth.

## Discussion

The current study has provided new insight into the interaction between *L. pneumophila* and mucin, which heretofore had been minimally investigated. Most notably, we documented that clinical isolates of *L. pneumophila* can utilize mucin as a food source. Although we had tried before to explore the growth-stimulating effect of mucin on *L. pneumophila* [42], we had (i) added 0.05 or 0.1 mg ml^−1^ of a mucin preparation into the CDM broth rather than 5 mg ml^−1^, (ii) used type II mucin (Sigma-Aldrich, catalogue #M2378), which is a crude preparation of mucin, from which the type III mucin used here is derived [92]. Also, we had previously employed starting bacterial inocula of ~1×10^8^ c.f.u. ml^−1^ rather than ~1×10^5^ c.f.u. ml^−1^ used here, and high cell densities suppress the expression of some of the factors needed for mucin utilization, e.g. some T2SS substrates are down-regulated in denser cultures during the stationary phase [8]. Thus, there are multiple possible reasons for the prior negative results. Importantly, however, the source (i.e. Sigma-Aldrich) and amount of mucin (i.e. 2.5 mg ml^−1^ for CDM agar or 5 mg ml^−1^ for CDM broth) that we currently utilize are identical or comparable to what has been employed to document mucin usage by many other bacteria [70,73,75]. Since the stimulation of *L. pneumophila* growth associated with added mucin was evident when iron was included in either the broth or agar media, it was not simply due to the iron that can sometimes occur in mucin preparations [70,73]. Although *L. pneumophila* was historically thought to only utilize amino acids as its carbon and energy source, newer studies have shown that the bacterium can also use sugars [93,94]. Hence, we surmise that the growth simulation we have observed is due to *L. pneumophila* assimilating peptides, amino acids and/or sugars released by the degradation of the mucin glycoprotein.

As was noted above, the mucin preparation used here contains glycoproteins that are present in the extracellular spaces in the lungs, and therefore, we strongly suspect that mucin utilization contributes to *L. pneumophila*’s ability to infect the respiratory tract and to cause disease. Although research into *L. pneumophila* pathogenesis has tended to focus on bacterial growth within macrophages, the fact that *L. pneumophila* has an extracellular phase within the lungs is indicated in various studies [95,98]. Interestingly, there is evidence that extracellular *L. pneumophila* may even induce airway epithelia to secrete mucins [99]. Finally, mucin degradation and utilization would presumably be a virulence determinant in rare cases of *L. pneumophila* infecting the human intestinal tract [100]. Overall, *L. pneumophila* joins a growing list of pathogens that can use mammalian mucins for their growth, with the other lung pathogens including *Acinetobacter baumannii*, *Burkholderia cepacia*, *Pseudomonas aeruginosa* and *Streptococcus pneumoniae* [52,70, 73, 74, 101, 102].

The second major conclusion of the current study is that *L. pneumophila*’s ability to optimally grow in mucin-containing media is mediated, to a great extent, through the action of a T2SS, ascribing yet another role to the *L. pneumophila* T2SS [8,24, 25]. Given the impaired ability of the *chiA* mutant and the *proA* mutant to grow in the presence of mucin, we infer that ChiA and ProA explain, at least in part, the role of the T2SS in mucin utilization by *L. pneumophila*. Since mucin is a large glycoprotein, it makes sense that multiple types of secreted enzymes would contribute to the utilization process. Although purified ChiA has been shown to degrade mucin [42], more work is needed to discern whether ProA directly degrades mucin. Since ProA degrades a wide range of host proteins, including collagen, vitronectin, antitrypsin, cytokines and complement components [96], it likely also acts on mucin. However, because ProA also cleaves and thereby activates other T2SS-dependent exoenzymes [41,103], it is also possible that ProA indirectly promotes mucin utilization by activating another mucin-degrading enzyme(s). Given the conservation of both the genes encoding the T2SS and the genes for ChiA and ProA in *L. pneumophila* genomes, including those of strains Philadelphia-1, Paris, Lens, Corby and Alcoy [8,35, 104], as well as Togus-1 (ORF 02142 for *chiA* and ORF 00464 for *proA*), the observations made here using strain 130b likely apply to many, if not all, *L. pneumophila* strains. Because the *lspF* mutant lacking the entire T2SS was more impaired for growth in the mucin-containing media than were the *chiA* mutant and *proA* mutant, we strongly suspect that additional T2SS substrates (unconnected to ChiA or ProA) are involved in *L. pneumophila*’s ability to use mucin. Candidate effectors include another chitinase-like protein and many other types of putative proteases that we recently confirmed as being T2SS substrates [25]. That a T2SS is involved in *L. pneumophila* mucin degradation/utilization is reminiscent of what has been documented for pathogenic *Escherichia coli*, *P. aeruginosa* and *Vibrio cholerae* [9,89,91]. The fact that the *lspF* mutant grew, albeit to a relatively small degree, in the presence of added mucin suggests that factor(s) in addition to the T2SS can promote the ability of *L. pneumophila* to grow in mucin-containing media. Although the identity of these factors is speculative, the possibilities include (i) other mucin-degrading exoenzymes that may be secreted via the type IV pilus apparatus, which mediates chitinase secretion by *Francisella novicida* [105] or the type I secretion system, which promotes the secretion of a mucin-binding protein by *Salmonella* spp. [106]; (ii) mucin-degrading enzymes that might operate within the outer membrane, analogous to the Sus-like system of *Bacteroidetes* species [107]; or (iii) the membrane transporters that might import mucin breakdown products that are already present in the medium, independent of the action of any microbial enzymes.

While examining bacterial growth on the various agar media, we fortuitously discovered that WT *L. pneumophila* exhibits increased spreading when grown on CDM agar vs. on BCYE agar. Since this phenotype was not observed for a mutant lacking surfactant [17,86], it reflects increased sliding motility, which is a form of passive movement that does not require flagellar or pilus appendages [54,108]. On the one hand, these data suggest that *L. pneumophila* surfactant production and its associated motility are more highly induced under lower-nutrient conditions or when the (main) food source is free amino acids (as in CDM). On the other hand, since the enhanced spreading of the WT legionellae did not occur when the CDM agar had added mucin, sliding motility is repressed by mucin or a mucin byproduct(s). That mucin or mucin-derived factors modulate other types of bacterial phenotypes has been observed for *A. baumannii*, *Bacillus cereus*, *P. aeruginosa*, *S. pneumoniae*, *V. cholerae* and elsewhere [50,70, 74, 75, 109,111]. Another current observation that warrants future inquiry is understanding why the T2SS mutant sometimes showed decreased recoverability in the base CDM broth, since T2SSs have generally not been linked to survival in (defined) media composed of free amino acids, metals and salts, i.e. lacking substrates for typical degradative exoenzymes [9]. One possibility, among many, is that a surface-localized T2SS substrate promotes the import of amino acids or metals or that the T2SS stabilizes the cell envelope under nutrient-limiting conditions.

Interestingly, enhanced growth in the presence of mucin was exhibited by only 3 out of the 12 other *Legionella* species examined, indicating that mucin utilization may be limited to a minority of *Legionella* species and that the species showing increased growth on the mucin-containing CDM agar, i.e. *L. feeleii*, *L. spiritensis* and *L. wadsworthii*, encode the T2SS, T2SS substrate ProA and, in the case of *L. feeleii* and *L. wadsworthii*, T2SS substrate ChiA [8], which is compatible with those three factors having a role in mucin utilization, as documented from our mutant analysis of *L. pneumophila*. However, since all of the other species that did not show growth or enhanced growth on the mucin-containing media also encode the T2SS, ProA and, in four cases (i.e. *L. anisa*, *L. bozemanii*, *L. cardiaca* and *L. lansingensis*), ChiA [8,112], the secretion system and these two exoenzymes are not sufficient for conferring mucin utilization. This also aligns with the results obtained from testing *L. pneumophila* mutants, which indicated that factors in addition to the T2SS contribute to mucin usage.

In conclusion, we have shown that *L. pneumophila* can utilize mucin for enhanced growth. We speculate that this process has a key role in lung infection. Thus, among other things, future work should discern how the various *L. pneumophila* exoenzymes degrade mucin, which mucin breakdown products are assimilated by *L. pneumophila* and how, when and where mucin degradation and assimilation occur during infection, and if and how mucin modulates sliding motility and perhaps other traits of *L. pneumophila*. It will also be instructive to test more (all) *Legionella* species for their ability to utilize mucin, to more fully document the degree of conservation of mucin utilization across the genus and ultimately gain additional insight into the factors, T2SS-related or otherwise, that allow legionellae to scavenge this nutrient. Finally, our development of CDM agar may prove useful as an alternative defined medium for studying other aspects of *L. pneumophila* metabolism and motility.

## Abbreviations

BCYE: buffered charcoal yeast extract
CDM: chemically defined medium
ChiA: chitinase
T2SS: type II secretion system
